# Revisiting Secondary Dilative Cardiomyopathy

**DOI:** 10.3390/ijms26094181

**Published:** 2025-04-28

**Authors:** Nilima Rajpal Kundnani, Federico Di Luca, Vlad Meche, Abhinav Sharma, Mihaela-Diana Popa, Marioara Nicula-Neagu, Oana Raluca Voinescu, Mihai Iacob, Daniel-Marius Duda-Seiman, Simona Ruxanda Dragan

**Affiliations:** 1 University Clinic of Internal Medicine and Ambulatory Care, Prevention and Cardiovascular Recovery, Department VI—Cardiology, “Victor Babes” University of Medicine and Pharmacy, 300041 Timisoara, Romania; knilima@umft.ro (N.R.K.); sharma.abhinav@umft.ro (A.S.); daniel.duda-seiman@umft.ro (D.-M.D.-S.); simona.dragan@umft.ro (S.R.D.); 2 Research Centre of Timisoara Institute of Cardiovascular Diseases, “Victor Babes” University of Medicine and Pharmacy, 300041 Timisoara, Romania; 3 Faculty of Medicine, “Victor Babes” University of Medicine and Pharmacy, 300041 Timisoara, Romania; di-luca.federico@student.umft.ro (F.D.L.); vlad.meche@umft.ro (V.M.); 4 Discipline of Microbiology, Department XIV Microbiology, “Victor Babes” University of Medicine and Pharmacy, 300041 Timisoara, Romania; 5 Physiology Discipline, Faculty of Bioengineering of Animal Resources, University of Life Sciences ”King Mihai I” from Timisoara, 300645 Timisoara, Romania; mnicula@animalsci-tm.ro; 6 Department of Cardiology, “Victor Babes” University of Medicine and Pharmacy, 300041 Timisoara, Romania; voinescu.oana@umft.ro

**Keywords:** dilative cardiomyopathy, precision medicine, biomarkers, targeted therapies

## Abstract

Secondary dilated cardiomyopathy (DCM) refers to left ventricular dilation and impaired systolic function arising from identifiable extrinsic causes, such as ischemia, hypertension, toxins, infections, systemic diseases, or metabolic disorders. Unlike primary DCM, which is predominantly genetic, secondary DCM represents a diverse spectrum of pathophysiological mechanisms linked to external insults on myocardial structure and function. The increasing prevalence of conditions such as alcohol use disorder, chemotherapy-induced cardiotoxicity, and viral myocarditis underscores the need for heightened awareness and early recognition of secondary DCM. A comprehensive analysis of clinical trial data and observational studies involving secondary dilative cardiomyopathy was conducted, with a focus on mortality, symptom relief, and major adverse events. A systematic literature review was performed using databases, including PubMed, Embase, and ClinicalTrials.gov, following PRISMA guidelines for study selection. Data were extracted on patient demographics, etiology of dilation, trial design, outcomes, and follow-up duration. Advances in diagnostic modalities have refined the ability to identify underlying causes of secondary DCM. For example, high-sensitivity troponin and cardiac magnetic resonance imaging are pivotal in diagnosing myocarditis and differentiating it from ischemic cardiomyopathy. Novel insights into toxin-induced cardiomyopathies, such as those related to anthracyclines and immune checkpoint inhibitors, have highlighted pathways of mitochondrial dysfunction and oxidative stress. Treatment strategies emphasize the management of the causing condition alongside standard heart failure therapies, including RAAS inhibitors and beta-blockers. Emerging therapies, such as myocardial recovery protocols in peripartum cardiomyopathy and immune-modulating treatments in myocarditis, are promising in reversing myocardial dysfunction. Secondary DCM encompasses a heterogeneous group of disorders that require a precise etiological diagnosis for effective management. Timely identification and treatment of the underlying cause, combined with optimized heart failure therapies, can significantly improve outcomes. Future research focuses on developing targeted therapies and exploring the role of biomarkers and precision medicine in tailoring treatment strategies for secondary DCM.

## 1. Introduction

Cardiomyopathies are diseases that affect the heart muscle, leading to changes in the structures and functions of the heart [1,2]. This review aims to focus on secondary dilated cardiomyopathy (DCM), a complex disease involving the heart muscle and characterized by left ventricular dilatation and systemic dysfunction, in the absence of other abnormalities that can be considered as a burden, such as ischemic heart disease or hypertension [3]. According to the American Heart Association, DCM can be classified into genetic/primary, mixed, or secondary forms [4,5,6].

Secondary dilated cardiomyopathy (DCM) is a myocardial disorder marked by ventricular dilation and impaired systolic function, resulting from an identifiable external cause rather than primary myocardial disease. This condition often arises as a complication of systemic illnesses, toxins, or metabolic abnormalities, distinguishing it from idiopathic or genetic forms of DCM [7]. Understanding secondary DCM is crucial due to its rising incidence and diverse etiological factors, including prolonged hypertension, chronic ischemia, and systemic inflammatory diseases such as sarcoidosis or systemic lupus erythematosus [8]. The condition is frequently misdiagnosed, leading to delays in appropriate intervention and worse patient outcomes. The current literature emphasizes the importance of early identification of causative factors and individualized treatment approaches, as secondary DCM may improve or even reverse with proper management of the underlying disease. Recent advances in molecular diagnostics and imaging technologies have enhanced the understanding and clinical management of secondary DCM, highlighting a need for further research into its complex pathophysiology and treatment strategies [9].

Although DCM can be genetic in origin, there are many secondary causes that can lead to the development of this disease, secondary causes that are good to know about, as once diagnosed and treated, they can lead to a significant improvement in prognosis. Such causes may include metabolic, infectious, toxic, or genetic factors [1,2,3,4,5,6,10,11,12].

The diagnosis of secondary DCM requires a multidisciplinary approach involving a thorough history, laboratory tests, and cardiac imaging. In addition, in selected cases, especially in suspected post-infectious myocarditis, a myocardial biopsy may provide useful information for diagnosis. The severity of secondary DCM further increases by the high risk of evolution into heart failure (HF) and sudden death. DCM is a major indication for heart transplantation, an unfavorable condition in which the survival rate is significantly reduced [13,14,15].

This review specifically aims to analyze secondary dilated cardiomyopathy and the benefits that early and targeted diagnosis and personalized treatments can offer in patient prognosis.

## 2. Epidemiology

The epidemiology of secondary dilated cardiomyopathy (DCM) shows a significant prevalence in the general population, with variations according to the populations examined and the diagnostic criteria applied. In particular, secondary DCM tends to be more frequent in tropical regions and less developed countries than in industrialized countries [16]. Secondary DCM contributes significantly to the global burden of heart failure, representing 30–50% of DCM cases in clinical practice [9]. Its prevalence varies with regional disparities in the incidence of underlying conditions. For instance, chronic ischemic heart disease and uncontrolled hypertension dominate as primary causes in developed countries, while infectious causes like Chagas disease are prevalent in endemic areas of Latin America. Among hospitalized heart failure patients, secondary DCM accounts for an estimated 10–20%, often associated with delayed diagnosis of the causative condition [17].

In industrialized countries, secondary DCM is commonly linked to chronic conditions, such as hypertension and coronary artery disease. In fact, research conducted in the United States showed an incidence of 0.56 cases per 100,000 population, with a large proportion of patients having comorbidities such as diabetes [18].

The risk of developing secondary DCM is notably higher in populations exposed to specific triggers, such as chemotherapy agents like anthracyclines, prolonged alcohol abuse, or systemic inflammatory diseases. Additionally, advanced age and the male sex are recognized as predisposing factors. Despite advances in diagnostic and therapeutic strategies, secondary DCM remains associated with high morbidity and mortality due to the multifactorial nature of its pathogenesis

Regarding so-called “idiopathic” dilative cardiomyopathy, an interesting study was conducted in Olmsted County, Minnesota, between 1975 and 1984, and it showed a rapid increase in DCM cases. This research, conducted on about 45 patients in Olmsted County, found that the disease incidence had doubled. During the first 5 years, the incidence was about 3.9 cases per person-years, and in subsequent 5-year intervals it rose to approximately twice that rate, at ca. Moreover, more than one-third of patients were diagnosed in NYHA class III or IV, which indicates a marked worsening of cardiac function [19].

There are also several cases related to nutritional deficiencies, including vitamin D, that influence the incidence of DCM. A clinical case in Ethiopia reported the case of an infant with heart failure induced by hypocalcemia, caused by maternal vitamin D deficiency, thus suggesting that nutritional deficiencies may also have a significant impact on cardiac function in developing countries [20].

Socioeconomic differences within the same country also affect the occurrence of DCM. Indeed, in some low-income communities, there is limited access to nutritious foods and medical care, thus leading to the development of this condition [20].

These differences have made known the importance of a personalized clinical approach in both the prevention and treatment of DCM while applying locally specific treatment regimens and characteristics of different populations. In addition, with the centralization of health services and access to advanced technologies, early diagnosis and treatment of DCM have improved, leading to a reduction in associated mortality [21] (see Table 1 for reference).

## 3. Etiology

The etiology of dilated heart disease (DCM) is complex and multifactorial, encompassing both genetic and secondary causes. Due to this changing etiology, DCM can be classified into three main forms: genetic/primary, mixed or secondary/secondary, according to the American Heart Association (AHA), or as familial and non-familial form, according to the European Society of Cardiology (ECS) [6].

Secondary DCM encompasses a broad spectrum of etiologies, reflecting its dependence on external precipitating factors. The most common causes include chronic ischemic damage from coronary artery disease and sustained hypertension, which lead to ventricular remodeling and impaired contractility. Toxins such as alcohol, cocaine, and chemotherapeutic agents induce myocardial injury through direct cytotoxic effects or oxidative stress pathways. Metabolic conditions, including hypothyroidism, hyperthyroidism, and diabetes mellitus, also play significant roles by altering myocardial energetics and calcium handling [22].

Idiopathic forms, according to several studies, are those with the highest incidence, with approximately 68% of DCM cases having an unknown origin, whereas viral infections, particularly myocarditis, have been identified as a causative etiological agent [12].

Systemic inflammatory conditions like sarcoidosis, lupus, and myocarditis further contribute to secondary DCM by provoking immune-mediated myocardial damage [23]. Emerging evidence highlights the role of infectious agents, including viral myocarditis and Chagas disease, which are prominent in specific geographical regions [24]. Furthermore, genetic predisposition may influence susceptibility to certain triggers, as seen in familial forms of toxin- or stress-induced cardiomyopathies [25]. Elucidating these diverse etiological pathways remains pivotal in tailoring treatment strategies to improve outcomes for affected individuals.

Table 2 shows the major etiological classes of secondary dilated cardiomyopathy, with their respective definitions, common causes, diagnostic tools, and estimated incidence.

## 4. Secondary Dilated Cardiomyopathy

Secondary dilated cardiomyopathy (DCM) is a condition where dilatation and left ventricular dysfunction are due to secondary causes rather than primary genetic mutations. Several causes are linked to secondary DCM, which are subdivided into infectious, toxic, endocrine, nutritional, and autoimmune. Among secondary causes of DCM, one of the most common is viral infections, in particular, myocarditis caused by coxsackievirus. These infections may damage the heart muscle to such an extent that cardiomyopathy worsens progressively [26]. Moreover, the abuse of toxic substances, e.g., alcohol, and some chemotherapeutics, for instance, can become a catalyst for DCM. Alcohol, specifically, induces a dilated cardiomyopathy through the mechanisms of direct toxicity and malnutrition [26]. Endocrine disorders, such as hypothyroidism and hypocalcemia, are other important causes of secondary DCM. Hypothyroidism may induce a balanced reduction of the heart and left ventricle dilation; meanwhile, hypocalcemia, most of the time, is related to hypoparathyroidism and can cause a reversible type of DCM [27,28]. An interesting case of DCM has been documented where it was linked to a selenium deficiency that occurred after bariatric surgery, thus underlining the role of proper nutrition in maintaining good cardiac health [29]. In addition, autoimmune diseases, like systemic lupus erythematosus, can inflame cardiac muscle and cause DCM. Such cases may trigger an immune response, damaging the cardiac tissue and worsening the condition eventually, which leads to ventricular expansion and dysfunction [26]. Finally, it is crucial to mention that secondary DCM might further arise due to systemic conditions, e.g., chronic kidney disease, which can impact the heart’s performance, through mechanisms of volumetric overload as well as electrolyte abnormalities. Early diagnosis and finding out the underlying cause are the cornerstone for the successful management of secondary DCM. Therefore, if the primary disease is treated, improvement in the heart’s function can be achieved [4,5].

## 5. Toxic Cardiomyopathy

Dilated cardiomyopathy secondary to exposure to toxic substances is one of the leading forms of secondary cardiomyopathy, where the change in health status, such as cardiovascular injury, is due to exposure to substances like toxins, drugs, or chemicals. The toxic causes of DCM can be divided into different categories, which include the use of drugs, exposure to chemicals, and substance abuse. One of the principal drug groups linked with toxic DCM is chemotherapeutic agents, especially anthracyclines, such as doxorubicin. These medications have been associated with cardiotoxicity, which is either acute or chronic DCM, depending on the dose and duration of the treatment. Cardiomyopathy induced by doxorubicin is due to oxidative damage, apoptosis, and mitochondrial dysfunction, which are known side effects of these medications [30]. Pediatric patients are considered to be the susceptible group to these drug-related cardio-toxic effects. Thus, they are at greater risk of developing DCM [31]. Other types of drugs, particularly some azole fungicides, have been tested for the potential of inducing cardiotoxicity, although the specific mechanisms are sometimes intractable to know. The alteration in cardiac gene expression and subsequent oxidative stress that these drugs might cause are factors that contribute to cardiac dysfunction.

Additionally, the use of substances such as alcohol and illicit drugs, like cocaine and amphetamines, is considered to be a significant risk factor for the development of DCM. Cardiomyopathy secondary to alcoholism is termed alcohol cardiomyopathy and is marked by progressive ventricular dilation and systolic dysfunction [32]. The tendency to have DCM is also associated with the ingestion of heavy metals, such as lead and cobalt. Although it is not always clear in the case of occupational exposure to metals and DCM, several researchers have suggested that these two are related, especially in the case of cobalt oxide, which has been shown to cause heart ailments [32]. Likewise, the substances, for example, carbon monoxide, can still be the cause of this type of cardiomyopathy through direct damage to the myocardium. Furthermore, the influence of toxic agents in the cosmetic and non-medical areas on the human body also needs to be considered. Examples include inhalation of hydrocarbons and solvents that depress the central nervous system and thus predispose toward arrhythmia and heart failure [33]. TCM diagnosis involves taking a careful history of toxin exposure and medication use, as well as managing the underlying causes of these patients.

## 6. Chemotherapy-Induced Cardiomyopathy

Chemotherapy-induced cardiomyopathy (CIC) is a significant clinical concern, particularly as cancer survival rates improve. Secondary dilated cardiomyopathy (DCM) resulting from chemotherapy is characterized by ventricular dilation and impaired systolic function, leading to heart failure symptoms. Understanding the pathophysiological mechanisms underlying CIC is crucial for early detection, prevention, and management.

Anthracyclines, such as doxorubicin, are among the most well-known chemotherapeutic agents associated with cardiotoxicity. Their cardiotoxic effects are dose-dependent and cumulative, with the risk of DCM increasing significantly at higher cumulative doses [34]. The primary mechanisms include the following:

- Oxidative Stress: Anthracyclines undergo redox cycling, producing reactive oxygen species (ROS) that overwhelm the heart’s antioxidant defenses, leading to lipid peroxidation, mitochondrial damage, and apoptosis of cardiomyocytes [35];
- Topoisomerase IIβ Inhibition: Doxorubicin interferes with topoisomerase IIβ in cardiomyocytes, causing DNA double-strand breaks and triggering cell death pathways [36];
- Mitochondrial Dysfunction: The accumulation of anthracyclines in mitochondria disrupts electron transport chains, leading to energy depletion and further ROS production [37].

Immune checkpoint inhibitors (ICIs), such as nivolumab and pembrolizumab, have revolutionized cancer therapy but are associated with immune-related adverse events, including myocarditis. ICI-induced myocarditis is characterized by T-cell infiltration of the myocardium, leading to inflammation, myocyte necrosis, and subsequent DCM. This condition often presents early in the treatment course and carries a high mortality rate if not promptly recognized and treated [38].

Other chemotherapeutic agents implicated in CIC include the following:

- Trastuzumab: A monoclonal antibody targeting HER2 receptors, trastuzumab disrupts cardiomyocyte survival pathways, leading to reversible cardiac dysfunction [39];
- Cyclophosphamide: An alkylating agent that can cause endothelial damage, leading to hemorrhagic myocarditis and subsequent DCM [40];
- 5-Fluorouracil (5-FU): An antimetabolite associated with coronary vasospasm and myocardial ischemia, potentially leading to DCM [41];
- Bevacizumab: An anti-VEGF agent that can induce hypertension and thromboembolic events, contributing to cardiac dysfunction [42].

## 7. Myocarditis, Inflammatory Cardiomyopathies, and Dilative Cardiomyopathy

Myocarditis is an inflammation of the cardiac muscle that can result from viral infections, autoimmune responses, or toxic exposures. This condition can also develop into dilated cardiomyopathy (DCM) because of its potential to cause structural and functional damage to the myocardium. The etiology of DCM secondary to myocarditis is complicated and involves several pathogenic mechanisms. One of the main mechanisms by which myocarditis can lead to DCM is chronic inflammation. During the acute phase of myocarditis, the inflammation may cause myocardial cells to die, resulting in necrosis and fibrosis. These processes can change the normal architecture of the heart and dilate the left ventricle, and the effectiveness of contraction is decreased [33]. Studies have shown that patients who survive an acute episode of myocarditis may develop chronic cardiac alterations, such as DCM, due to scar formation and ventricular remodeling [33]. Furthermore, the persistence of infectious agents, like cytomegalovirus, can play a role in chronic inflammation leading to DCM. Persistent viremia accompanied by ineffective ones will trigger a long-lasting myocardial injury, which will cause a progressive reduction in heart function [43]. In one documented case, a patient with cytomegalovirus myocarditis showed an improvement in left ventricular function after antiviral treatment. Thus, infection control may be the key to preventing further complications of DCM [44]. Another aspect is the role of autoimmunity in the pathogenesis of DCM secondary to myocarditis. In some patients, myocardial inflammation might drive an autoimmune response, in which the immune system mistakenly attacks the cardiac cells, thus furthering the malfunction of the cardiac system and ventricular dilation [45]. This mechanism has been observed in patients with myocarditis associated with autoimmune diseases, where the markers of inflammation and autoimmunity were found to be elevated [45].

Recent research has further elucidated the immunological and molecular features of inflammatory cardiomyopathies that contribute to secondary DCM. According to Lauriero et al. [46], inflammatory cardiomyopathy represents a distinct pathophysiological entity, often following viral myocarditis but sometimes persisting without detectable viral genomes. In these cases, inflammation is driven predominantly by immune dysregulation rather than ongoing infection. The European Society of Cardiology Working Group [3] emphasizes that chronic inflammation, particularly with macrophage and T-lymphocyte infiltration, can sustain myocardial dysfunction even in the absence of acute symptoms.

Cardiac magnetic resonance (CMR) imaging and endomyocardial biopsy (EMB) are crucial in diagnosing inflammatory cardiomyopathies. These modalities help distinguish between active myocarditis, healed myocarditis with residual inflammation, and non-inflammatory DCM. This diagnostic precision has therapeutic implications, especially in cases where immunosuppressive therapy may benefit patients with virus-negative, immune-mediated myocarditis.

In addition, the role of genetic predisposition in the progression from myocarditis to DCM is gaining attention. According to Trachtenberg et al. [47], certain HLA alleles are associated with susceptibility to myocarditis and the development of autoimmune-mediated myocardial damage. This supports a model in which environmental triggers such as infections initiate inflammation in genetically predisposed individuals, potentially resulting in persistent autoimmunity and myocardial remodeling.

Therapeutically, managing inflammatory cardiomyopathy remains challenging. Antiviral therapies have shown benefit in virus-positive myocarditis, while immunosuppressive regimens—particularly corticosteroids and azathioprine—are increasingly used in virus-negative, immune-mediated cases [48]. Targeted immunomodulatory therapy is under investigation, aiming to selectively suppress autoreactive lymphocytes while preserving host defense mechanisms.

The identification of circulating biomarkers, such as soluble ST2, troponins, and inflammatory cytokines, may aid in monitoring disease activity and guiding treatment, although no single marker has yet proven definitive [49]. As research advances, the goal is a more personalized approach to therapy, integrating molecular diagnostics, imaging, and immune profiling to better manage and potentially reverse inflammatory DCM progression.

Lastly, myocarditis can be caused by bacterial and parasitic infections, such as the case of Chagas’ disease, which is the main cause of non-ischemic cardiomyopathy in Latin America. In this case, the *Trypanosoma cruzi* infection causes myocarditis, and the patient can become a long-term DCM case. The disease progression is often characterized by progressive myocardial damage and significant morbidity and mortality.

## 8. Peripartum Cardiomyopathy

Peripartum cardiomyopathy (PPCM) is a form of dilated cardiomyopathy that is observed during pregnancy or a few months immediately after delivery. Left ventricular systolic dysfunction is a key characteristic of this condition, and its etiology remains mostly uncertain. However, it is thought that genetic, hormonal, and inflammatory factors may play the main part [50,51]. PPCM is classified as a type of secondary cardiomyopathy, as it is related to the physiological and hormonal stress that a woman goes through during pregnancy. The most glaring among the convincing reasons in PPCM is its resemblance to idiopathic dilated cardiomyopathy. Studies have shown that certain types of PPCM alone might be part of a continuum with some types of familial dilated cardiomyopathy. This fact has drawn attention to a probable common genetic background [52]. Additionally, it was found that women who have a family history of dilated cardiomyopathy may be at a relatively higher risk of developing PPCM, potentially due to genetic factors, indicating the possibility of genetic factors being responsible for their predisposition to the said condition [52]. The pathophysiology of PPCM is intricate and may be associated with the activity of hormonal factors such as prolactin, which, when present in high levels, may be cardiotoxic. The scientific evidence indicates that it is a fragment of prolactin that is cardiotoxic and may therefore be responsible for the myocardial dysfunction observed in PPCM [50]. Also, oxidation and oxidative stress have been noted as the main contributors to the disease pathway, thus indicating that inflammation and oxidative damage are partners in crime to the disease progression [52]. In the case of PPCM, the disease is usually hard to diagnose due to the symptoms that might overlap with usual pregnancy ones or with some cardiac conditions. The latency in diagnosis may consequently cause the worsening of the condition and result in higher mortality. The treatment of PPCM should be a team effort that would constitute the use of diuretics, inotropic drugs, and possibly bromocriptine, which has shown promising results in the recovery of left ventricular function [50]. Regarding prognosis, PPCM can present with a wide range of clinical outcomes, from one woman having preserved functioning of the heart to another female whose condition deteriorates to the level of heart failure or even complications with serious consequences during the pregnancy or in the post-partum period [50,53]. Thus, the treatment of PPCM must be individualized, which, in turn, implicates the doctor in tailoring the therapy for each particular case as far as the current situation and further pregnancies are concerned [50]. This indicates the need for a multidisciplinary approach.

## 9. Other Causes of Dilative Cardiomyopathy

Other causes of secondary DCM include conditions that can be secondary through different types of ailments: rheumatic fever, thyroid dysfunction, and sarcoidosis. Each of these has specific pathogenic mechanisms that may no less contribute to the incidence of DCM.

### 9.1. Rheumatic Fever

Rheumatic fever is an inflammatory consequence of a group A beta-hemolytic streptococcus-induced infection. Myocardial inflammation caused by rheumatic heart disease may evolve into DCM. Valve inflammation due to rheumatic fever may lead to damage to the myocardium, and its consequences would be fibrosis and ventricular dilation [54,55]. The study demonstrated that rheumatic fever was associated with a higher morbidity of heart diseases, particularly in patients who had had a previous history of rheumatic fever [54]. Furthermore, the trivia of the immunologic violence of the anti-streptococcus antibodies may manifest as a permanent myocardial inflammation, consequently leading to severe heart failure [55].

### 9.2. Endocrine-Related Dilative Cardiomyopathy

Endocrine disorders are the main cause of secondary DCM, which involves conditions like hypothyroidism, hyperthyroidism, and adrenocorticoid insufficiency, which exhibit a negative effect on the heart.

Hypothyroidism is marked by both reduced myocardial contractility and ventricular dilation. Studies have documented that the introduction of thyroid hormones in patients with DCM and hypothyroidism led to the improvement of cardiac function, which means that the correction of thyroid dysfunction is the key point in the management of DCM [56]. Moreover, low levels of T3 (free triiodothyronine) have been associated with a poor prognosis in DCM patients [57].

In contrast, hyperthyroidism may cause arrhythmia and overworking of the heart, hence, provoking heart dysfunction. Notably, suppression of hyperthyroidism in less than a week ensures prevention of thyroid-related cardiac pathology, which may exhibit symptoms typical of DCM [58]. Hyperthyroidism should be managed either through antithyroid drugs or other alternatives if the patient is not responsive to the first-line medications. Concomitant therapies aimed at improving cardiac function and quality of life should be considered.

Adrenal insufficiency can be linked to DCM. The lack of cortisol can lead to altered heart function, resulting in reversible cardiomyopathy in patients with adrenal insufficiency. Studies have documented that corticosteroids can improve cardiac function in patients suffering from adrenal insufficiency and DCM [56].

### 9.3. Sarcoidosis

Sarcoidosis is a systemic inflammatory disorder that causes granulomatous inflammation in various organs, including the heart. A possible consequent hazard is myocarditis and, in some instances, DCM. The granulomatous infiltration of the myocardium can contribute to cardiac dysfunction through the mechanisms of inflammation and fibrosis [59]. It has been shown in studies that sarcoidosis patients may experience the development of DCM, accompanied by a high rate of arrhythmias and heart failure [60]. The early diagnosis and cure of sarcoidosis are pivotal in directing the aim of prevention of VF.

### 9.4. Dyssynchronopathy: Left Bundle Branch Block-Induced Cardiomyopathy

Left bundle branch block (LBBB) is more than just a conduction abnormality; in certain patients, it can lead to a specific form of dilated cardiomyopathy known as dyssynchronopathy. This condition arises when the electrical delay caused by LBBB leads to mechanical dyssynchrony between the left and right ventricles. The resulting uncoordinated contraction impairs cardiac efficiency, promoting adverse remodeling and progressive left ventricular (LV) dysfunction [61].

Patients with LBBB-induced cardiomyopathy often present with symptoms of heart failure, such as dyspnea and fatigue. Diagnostic evaluation includes electrocardiography (ECG) to identify LBBB, echocardiography to assess LV function and mechanical dyssynchrony, and cardiac magnetic resonance imaging (MRI) to evaluate myocardial fibrosis and ventricular volumes [62].

The cornerstone of treatment is cardiac resynchronization therapy (CRT), which involves biventricular pacing to restore synchronous ventricular contraction. CRT has been shown to improve symptoms, enhance LV function, and reduce mortality in patients with LBBB and reduced ejection fraction. Studies have demonstrated significant improvements in LV ejection fraction and functional status following CRT implantation [63].

### 9.5. Atrial Fibrillation-Induced Dilated Cardiomyopathy

Atrial fibrillation (AF) is a common arrhythmia that, when persistent and associated with a rapid ventricular rate, can lead to a form of tachycardia-induced cardiomyopathy. The irregular and often rapid heart rate in AF impairs ventricular filling and reduces cardiac output, leading to LV dilation and systolic dysfunction over time [64].

Patients may present with symptoms of heart failure, including shortness of breath, fatigue, and exercise intolerance. Diagnosis involves ECG and Holter monitoring to detect AF and assess ventricular rate, as well as echocardiography to evaluate LV size and function.

Management focuses on controlling the heart rate and restoring sinus rhythm. Rhythm control strategies, including antiarrhythmic medications and catheter ablation, aim to restore and maintain normal heart rhythm [65]. Studies have shown that early rhythm control in patients with AF can reduce the risk of cardiovascular complications and improve outcomes. Catheter ablation, in particular, has been effective in reversing LV dysfunction in patients with AF-induced cardiomyopathy [66].

### 9.6. Premature Ventricular Complex-Induced Cardiomyopathy

Frequent premature ventricular complexes (PVCs) are often considered benign; however, a high burden of PVCs can lead to a reversible form of cardiomyopathy. The mechanism involves the disruption of normal ventricular activation and contraction patterns, leading to dyssynchronous myocardial contraction, impaired ventricular function, and eventual LV dilation [67].

Patients may be asymptomatic or present with palpitations, fatigue, or symptoms of heart failure. Diagnosis is established through Holter monitoring to quantify PVC burden and echocardiography to assess LV function.

Treatment aims to reduce PVC frequency and restore normal ventricular function. Catheter ablation has emerged as an effective therapy for patients with a high PVC burden and LV dysfunction, leading to significant improvements in LV ejection fraction and symptoms [68]. Antiarrhythmic medications may also be considered in certain cases. Studies have demonstrated that successful elimination of PVCs through ablation can result in the reversal of cardiomyopathy and normalization of LV function [69].

## 10. Diagnosis

The diagnosis of secondary dilated cardiomyopathy (DCM) requires an interdisciplinary approach and systematic work involving a careful history, a detailed clinical examination, laboratory tests, ECG, and advanced imaging techniques. The combination of different diagnostic methods makes it possible to identify the underlying cause of DCM or at least to rule out other possible causes.

### 10.1. Anamnesis and Clinical Examination

Getting a well-detailed anamnesis is imperative for determining the cause of secondary DCM. Data should be collected about a patient’s family history of heart disease, medication use, exposure to toxic substances, and pre-existing medical conditions, such as hypertension or diabetes, respectively [70]. The physical examination may therefore show the signs of heart failure, such as peripheral edema, lung crackles, and venous congestion. The presence of atrioventricular conduction defects or a family history of DCM may indicate a genetic susceptibility to the disease [70].

Laboratory tests play a vital role in the evaluation of cardiac function and diagnosing possible metabolic or endocrine causes leading to the development of DCM. Biomarkers such as B-type natriuretic peptide (BNP) and cardiac troponin can be used to assess the severity of cardiac dysfunction and the presence of myocardial damage, respectively [71]. Moreover, the creatine phosphokinase (CPK) level analysis is a valuable tool for monitoring inflammation or muscle injury [71]. The assessment of other conditions, such as hypothyroidism, is essential because it can result in reversible DCM [71].

### 10.2. Electrocardiogram (ECG)

The ECG is another diagnostic device indispensable in DCM evaluation. It can detect certain abnormalities in rhythm, conduction blocks, and ventricular hypertrophy, which might be secondary manifestations of DCM [72]. For instance, the ECG can show signs of ischemia or electrical changes that suggest a cardiomyopathy secondary to coronary heart disease or systemic conditions [72]. The combination of clinical findings, laboratory tests, and ECG can give a comprehensive diagnostic picture and facilitate the identification of the underlying causes of DCM.

Early studies have shown that these diagnostic methods are significant for secondary DCM. For example, a study demonstrated that clinical and laboratory analysis, along with ECG, are the crucial steps to preclude the secondary causes of DCM, such as endocrine and infectious diseases [73]. Moreover, the assessment of family history and the identification of genetic anomalies can enhance the diagnosis and management of the disease [14].

### 10.3. Imaging

Imaging techniques play a pivotal role in diagnosing secondary dilated cardiomyopathy (DCM), offering precise insights into structural and functional abnormalities. Transthoracic echocardiography (TTE) serves as the first-line modality due to its accessibility and ability to detect ventricular dilation, reduced systolic function, and secondary complications like mitral regurgitation. Advanced techniques such as speckle tracking echocardiography (STE) further refine diagnosis by assessing myocardial strain, which can detect subclinical disease and provide prognostic markers for left and right ventricular dysfunction [74].

Cardiac magnetic resonance imaging (CMR) is considered the gold standard for secondary DCM assessment, providing high-resolution evaluation of cardiac morphology, function, and tissue characteristics. It enables quantification of ventricular volumes, ejection fraction, and myocardial fibrosis using late gadolinium enhancement (LGE). Fibrosis detected through CMR imaging correlates strongly with adverse outcomes, including sudden cardiac death, even in patients with preserved ejection fraction [75]. Newer techniques like native T1 and extracellular volume (ECV) mapping offer detailed insights into interstitial remodeling, strengthening diagnostic accuracy and prognostic predictions.

Nuclear imaging and cardiac computed tomography (CT) play complementary roles in specific cases, such as identifying infiltrative processes or coronary artery disease contributing to secondary DCM [76]. Stress imaging modalities, including stress echocardiography, are useful for evaluating ischemia and functional reserve in patients with borderline findings or suspected ischemic contributions to the cardiomyopathy.

Hence, a multimodal approach combining echocardiography, CMR, and other advanced imaging techniques ensures accurate diagnosis and comprehensive management of secondary DCM. This allows tailored interventions, improved prognosis, and close monitoring of disease progression.

#### 10.3.1. Echocardiography

**Figure 1:**
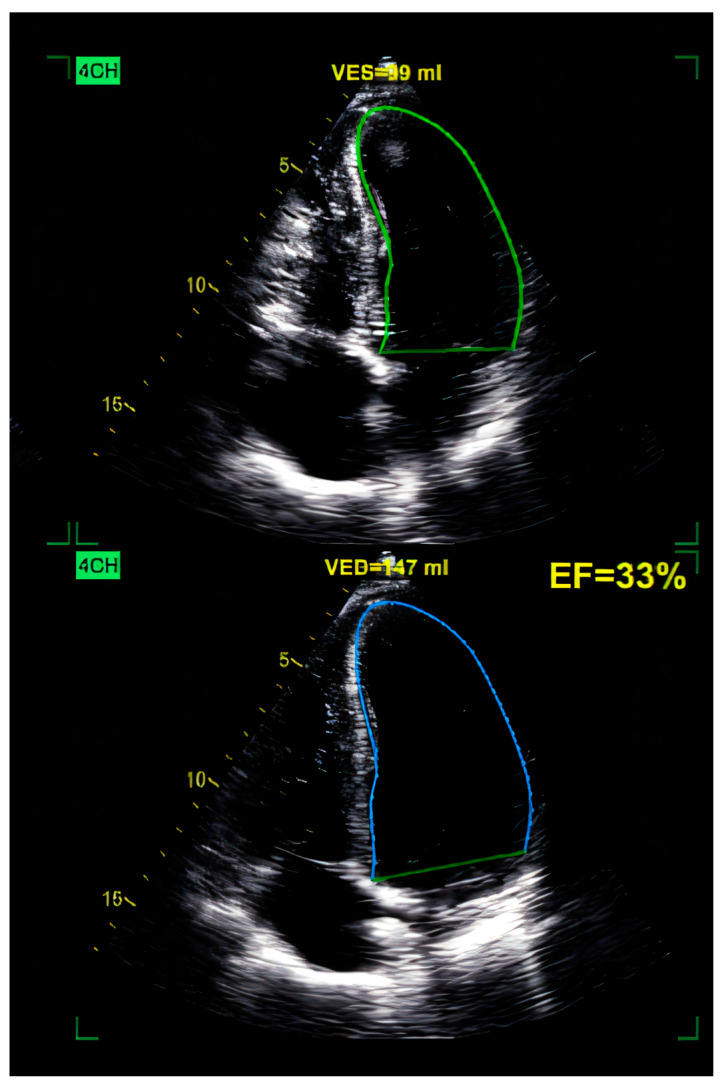
Two-dimensional TTE (transthoracic echocardiography) of the LV (left ventricle), revealing a dilated LV of 147 mL, with globally reduced EF of 33% and an apical thrombus (upper green area) in a patient with anterior MI. 4CH = apical 4-chamber view.

**Figure 2:**
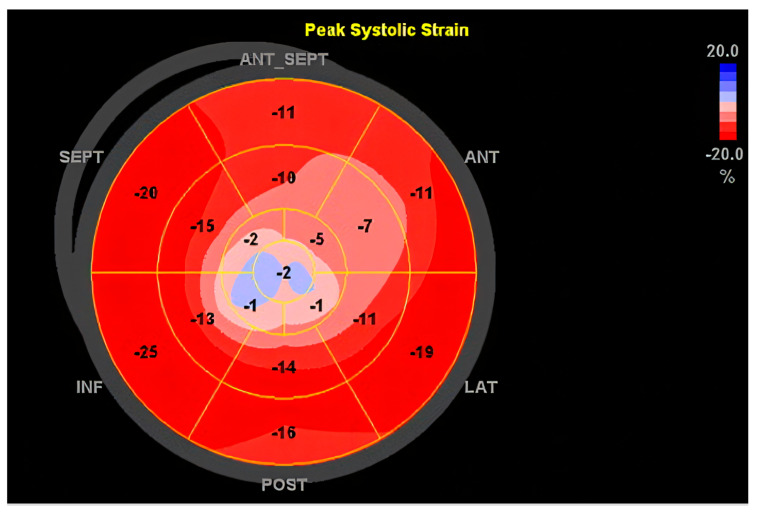
Speckle tracking of the LV, revealing reduced GLS (global longitudinal strain) of −12.4%, revealing the “blueberry on top” pattern. Values of regional strain higher than −18 show hypokinesia in the apical (center) segments of the left ventricle with akinesia in the LV apex revealed by the blue area on top. ANT_SEPT = antero-septal wall of LV, ANT = anterior wall of LV, LAT = lateral wall of LV, POST = posterior wall of LV, INF = inferior wall of LV, SEPT = septal wall of LV.

The echocardiography method, which has the highest accuracy in the diagnosis of dilated cardiomyopathy, is reserved for cases in which this cardiomyopathy is secondary to systemic infections, toxicity, or systemic disease. This technique is critical when it comes to the assessment of the volume (i.e., the heart’s pumping capacity), as well as to the diameter of the heart, thus enabling the suspicion that left ventricle dilation and systolic dysfunction are typical features of DCM [77]. Transthoracic echocardiography is a standard for diagnosing DCM as it is a non-invasive procedure that provides very comprehensive information on cardiac morphology and hemodynamic function [78]. Cardiac echo has proved to be highly beneficial in secondary DCM cases. For instance, one of the characteristics of echocardiography is its ability to reveal structural or functional abnormalities that might indicate the presence of DCM secondary to conditions such as hypertension or valvular diseases [79]. Further, the utilization of sophisticated echocardiography techniques like strain imaging has enhanced the evaluation of myocardial deformation, which in turn delivers prognostic information [77]. These advanced techniques may also distinguish DCM from other types of cardiomyopathy, such as that from ischemia, which requires exclusion via coronary angiography [80]. In Figure 1, we emphasize a 2D transthoracic echocardiography image of a patient with an anterior myocardial infarction (MI), revealing a dilated left ventricle (LV) of 147 mL with global reduced ejection fraction (EF), EF = 33%, with akinesia of apical segment of the LV, complicated with a thrombus (Figure 1). Speckle tracking evaluation revealed a reduced global longitudinal strain, predominantly in the apical segments of the LV, emphasizing the “blueberry on top” pattern (Figure 2).

**Figure 3:**
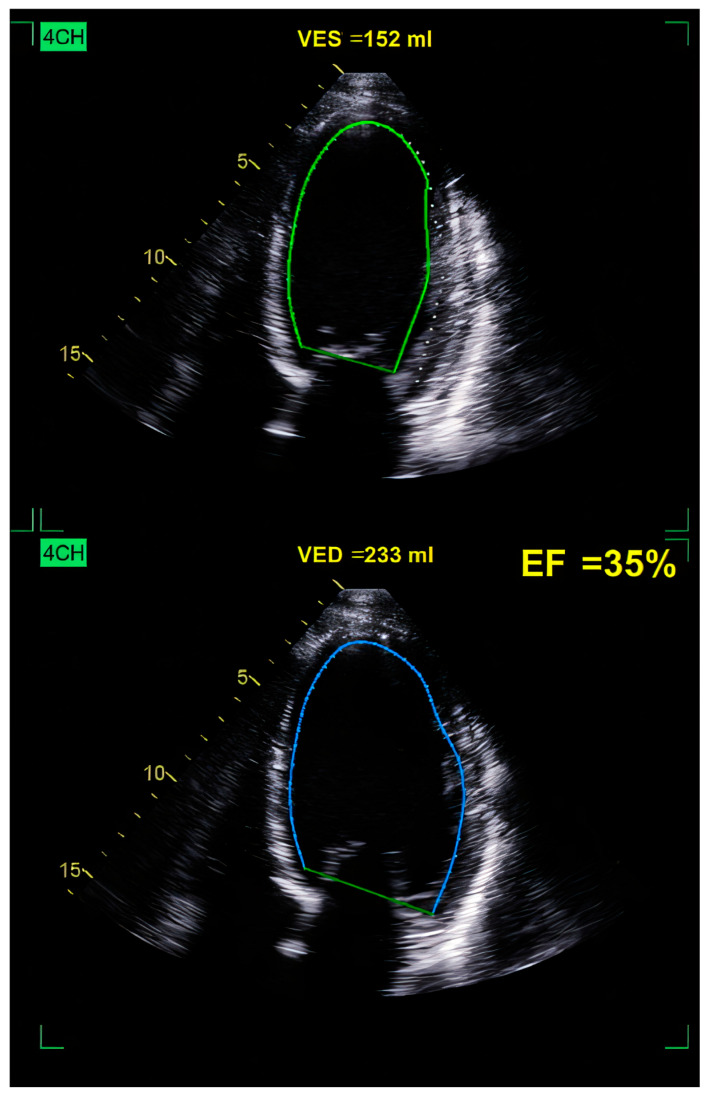
Two-dimensional TTE showing a dilated LV, with high VED of 233 mL, marked by the blue contour and increased VES of 152 ml emphasized by the green contour. Global LV hypokinesia and reduced EF (ejection fraction) of 35% in a patient with ethanolic DCM. VES = end-systolic volume of LV, VED = end-diastolic volume of LV, 4CH = apical 4-chamber view.

**Figure 4:**
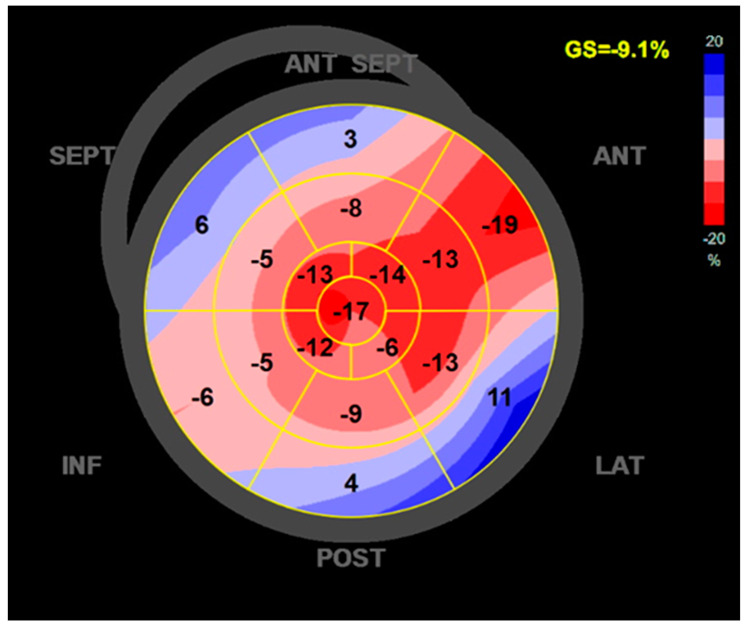
Reduced GLS of the LV of −9.1% in a patient with ethanolic DCM. Values of regional strain higher than −18 (pink, light blue, dark blue) show hypokinesia in the lateral, posterior, inferior, septal, and antero-septal regions of the left ventricle. ANT SEPT = antero-septal wall of LV, ANT = anterior wall of LV, LAT = lateral wall of LV, POST = posterior wall of LV, INF = inferior wall of LV, SEPT = septal wall of LV.

We emphasize the echocardiographic images of a patient with ethanolic DCM. Initial evaluation revealed a dilated LV with diffuse global hypokinesia and a reduced EF of 35% (Figure 3 and Figure 4). Optimal guide-directed heart failure treatment was initiated, and he abandoned alcohol consumption. TTE reevaluation showed V reverse remodeling with improvement in the LV volume and EF.

Cardiac troponins and some other biochemical markers, besides echocardiography, are usually employed to diagnose DCM as well as to determine the risk in patients [81]. The combination of these biomarkers with echocardiography might present a more comprehensible picture of the disease and might help clinicians in the management of the patients. The advent of artificial intelligence (AI) brings about great promises, as DCM diagnosis can become better by means of the automation of the echocardiography image analysis. The use of machine learning algorithms to analyze large volumes of echocardiogram data can point out patterns and abnormalities that are not immediately observable by the human eye [82]. The application of AI image analysis techniques could lead not only to a more precise diagnostic process but also to fewer delays in the interpretation of medical images, generating a correct diagnosis of DCM in a much faster manner. In summary, echocardiography remains the main diagnostic tool of DCM, especially for secondary cases, because of its ability to give detailed information regarding the structure and function of the heart. The integration of the use of other techniques and the application of AI solutions are two of the possible new developments that could improve the diagnosis and treatment of this complex disease.

#### 10.3.2. Cardiac Magnetic Resonance Imaging (MRI)

Cardiac magnetic resonance imaging (MRI) is the gold standard in the diagnosis of DCM, especially for the secondary forms. This imaging technique has the upper hand over other modalities in terms of detailed morphology and cardiac function, as well as information on the existence of myocardial fibrous tissue and other structural abnormalities. The ability of MRI to visualize the cardiac tissue non-invasively is extremely beneficial for detecting the underlying causes of DCM, such as systemic diseases or genetic mutations [83]. Recent studies have shown that MRI has the capability to detect structural and functional alterations in the myocardium that might not be as apparent with other imaging modalities such as echocardiography. For instance, MRI can thus recognize myocardial fibrosis by the use of specific sequences, like T1 mapping and late gadolinium imaging, which are the main aspects for the diagnosis and risk stratification in patients with DCM [84]. Furthermore, MRI is very much invaluable in the assessment of the secondary DCM, since it is able to spot abnormalities that may be linked to conditions such as hypertension or endocrine diseases [83]. The integration of artificial intelligence (AI) in MRI is being developed as a promising area for further enhancing the diagnosis of DCM. Machine learning algorithms can be applied to MRI images to detect patterns and abnormalities that might not be readily visible to the human eye. A case in point is the automated image segmentation techniques, which can facilitate the quantitative analysis of heart sizes and functions by increasing diagnostic accuracy and reducing interpretation time [84]. To sum up, MRI is the gold standard for DCM diagnostics, particularly in secondary forms, owing to its high resolution in cardiac structure and function. The integration of AI in MRI holds significant prospects to improve the diagnosis and management of DCM, thus facilitating a more accurate and timely analysis of cardiac images.

Of course, each of the aforementioned imaging techniques has its own strengths and limitations, as described in Table 3. A clinician must take into account the means available and tailor the diagnosis work-up based on each patient, individually.

#### 10.3.3. Future Directions: Integrating Artificial Intelligence in Imaging

Artificial intelligence (AI) is increasingly transforming cardiology by enhancing diagnostic accuracy, streamlining workflows, and supporting personalized treatment strategies. In the context of secondary dilated cardiomyopathy (DCM), AI offers promising advancements in both diagnosis and management.

AI-driven imaging analysis has shown significant potential in improving diagnostic precision. For instance, a study by Wolterink et al. demonstrated that convolutional neural networks could automatically segment cardiac structures in cine MRI and accurately classify various cardiomyopathies, including DCM, achieving a 91% accuracy rate. Such automation not only reduces inter-observer variability but also accelerates the diagnostic process [85].

In echocardiography, deep learning models like EchoNet-LVH have been developed to quantify ventricular hypertrophy and differentiate its etiologies with high precision. This model achieved a mean absolute error of 1.4 mm in measuring intraventricular wall thickness and demonstrated strong performance in classifying conditions such as hypertrophic cardiomyopathy and cardiac amyloidosis. These advancements facilitate early detection and tailored treatment plans for patients with secondary DCM [86].

Beyond imaging, AI tools are being explored for their potential in clinical decision support. The integration of AI into electronic health records can assist clinicians in identifying patients at risk, suggesting evidence-based interventions, and monitoring treatment responses. As highlighted in a review by Madaudo et al., AI applications, including language models like ChatGPT, are being considered for their role in patient education and clinical documentation, further enhancing patient care [87].

Incorporating AI into the diagnostic and therapeutic pathways of secondary DCM holds the promise of improved patient outcomes through more accurate diagnoses, personalized treatment strategies, and efficient healthcare delivery. Ongoing research and clinical validation are essential to fully realize the benefits of AI in this domain.

## 11. Therapy

The treatment of dilated cardiomyopathy (DCM) includes a combination of pharmacological and surgical approaches aimed at improving symptoms, preventing disease progression, and reducing morbidity and mortality.

Pharmacological therapy forms the cornerstone of management. Angiotensin-converting enzyme inhibitors (ACEIs) or angiotensin receptor blockers (ARBs), alongside beta-blockers, are first-line therapies that improve left ventricular function and reduce mortality [88]. Mineralocorticoid receptor antagonists (MRAs) are added for patients with persistent symptoms or in those having heart failure with reduced ejection fraction (HFrEF) [89]. In recent years, sodium-glucose cotransporter 2 inhibitors (SGLT2is) have emerged as effective agents for heart failure management, offering benefits in both diabetic and non-diabetic DCM patients [90]. Diuretics are prescribed for symptomatic relief in cases of volume overload, though they do not impact disease progression [91].

Surgical interventions are considered in advanced or refractory cases. Implantable cardioverter-defibrillators (ICDs) are recommended for patients at risk of sudden cardiac death due to ventricular arrhythmias, while cardiac resynchronization therapy (CRT) improves outcomes in those with asynchrony [92]. In severe cases of DCM with end-stage heart failure, left ventricular assist devices (LVADs) or heart transplantation provide lifesaving options [93]. Surgical correction of mitral regurgitation through annuloplasty or valve replacement may also be necessary if secondary valve dysfunction contributes significantly to heart failure symptoms [94].

Emerging therapies, including gene-targeted treatments and regenerative medicine approaches such as stem cell therapy, hold promising results for treating the underlying causes of genetic or secondary DCM [95]. Multidisciplinary care is essential, integrating pharmacological, device-based, and surgical strategies to optimize outcomes and improve the quality of life.

### 11.1. Pharmacology

The pharmacological management of secondary dilated cardiomyopathy is of paramount importance for symptom control and enhancing cardiac function. Various clinical trials have proved the effectiveness of particular medicines that are adapted to the different causes of DCM.

One of the most widely studied methods is hormonal therapy for patients with DCM due to hypothyroidism. Studies have confirmed that thyroid hormone replacement can result in notable improvements in cardiac function. For example, one study has shown that DCM patients with hypothyroidism who have been treated with levothyroxine experienced a decrease in left ventricular dimensions and an increase in ejection fraction [96]. The result of this study proves that hypothyroidism treatment can directly lead to the reversal of DCM. Likewise, in patients with adrenal insufficiency, glucocorticoid therapy has been proven to improve cardiac function. Research reported that DCM in a patient with adrenal insufficiency who was treated with corticosteroids had complete recovery. Hormonal therapy has been used to help some patients, such as a patient with reversible DCM treated with corticosteroids in a clinical setting where adrenal insufficiency is thought to be underdiagnosed. These results underline the importance of hormonal levels for heart health [97].

Another important part of the pharmacological treatment in secondary DCM is the use of anticoagulants. Ventricular thrombi are often present in patients with DCM, and anticoagulation therapy has contributed to a decrease in thromboembolic events. Patients with DCM receiving anticoagulation have been shown to have fewer thromboembolic complications than those not treated with anticoagulants. This factor is critical to both the prognosis and the quality of life of these patients.

Finally, the management of secondary DCM usually includes drugs for blood pressure control and heart failure management, such as ACE inhibitors and beta-blockers. These medications are not only symptom-relieving but have additionally been linked to mortality reduction in patients with heart failure [98]. Using these treatments in conjunction with the therapies aimed at the underlying conditions brings up a comprehensive, integrated, and multidisciplinary approach to the management of secondary DCM.

Table 4 shows the main pharmacological treatments for secondary dilated cardiomyopathy, each with its own indications, strengths, and limitations.

### 11.2. Surgery

Secondary dilated cardiomyopathy (DCM) surgical treatments are one of the most effective methods for improving the patient’s condition and his/her quality of life. The surgical procedures include valve repair or replacement, removal of destroyed cardiac muscle, and, if appropriate, heart transplantation. The procedure of choice is the surgical approach based on the underlying cause of the disability and the severity of the disease. These include valve repair, valve replacement, and mitral valve repair in selected cases. Mitral valve repair is one of the most commonly performed procedures in patients with mitral insufficiency secondary to DCM, including the introduction of new methods of mitral valve repair. For example, one research work revealed that patients who underwent mitral valve repair were found to have a rise in ejection fraction, and heart failure symptoms had diminished [99]. Based on this finding, it is an advantage to pediatric populations where DCM mostly leads to valvular deformations and heart failure in the future, which can be avoided [100].

Furthermore, aortic valve replacement has been linked to favorable outcomes in patients with DCM secondary to aortic stenosis. It has been stated that individuals who underwent aortic valve replacement showed good cardiac function, and mortality rates were considerably reduced in the long term [101]. This particular treatment method is important in the case of ischemic DCM, where resolving valvular issues can improve the cardiac pumping capacity, enhancing the blood supply to the myocardium and the entire body. Heart transplantation is one of the options for patients with severe heart damage, where medical and other surgical options are inefficient. Studies have, by and large, proven that DCM patients who have undergone heart transplantation show better survival rates compared to those without [102].

Table 5 shows the non-pharmacological options for treating secondary DCM, along with their individual benefits and flaws.

### 11.3. Emerging Therapies

Emerging and experimental therapies for secondary DCM are offering new and original ways of dealing with this problem and thus improving the patient’s quality of life and prognosis. These therapies include cellular, pharmacological, and regenerative approaches, showing positive results, which are documented in the literature. One of the most promising areas is stem cell therapy. Research indicates that MSCs can reduce the fibrous tissues between cells and thus, help to improve cardiac functions in DCM models. Thus, umbilical cord-derived MSCs, according to a study done on a rat model of DCM, prevented the development of inflammation and consequently increased cardiac function due to the inhibition of TNF-α and TGF-β1/ERK1/2 signaling pathways [103]. Cellular therapies, thus, might be the best option for patients with severe DCM.

Moreover, gene therapy is also considered to be a possible method of treatment for DCM in the near future. Recent studies have shown that the transplantation of specific genes can improve heart function and slow down the progression of the disease. A newly found gene mutation was determined to be the cause of DCM in a child, which suggests that gene therapy might be a feasible way to treat the molecular basis of the disease [104]. These approaches aim to target the root causes of the disease and can be revolutionary in DCM treatment.

Innovative pharmacological therapies remain one of the areas in which research is being done. A meta-analysis of the effectiveness and safety of stem cell therapy in improving cardiac function and reducing heart failure symptoms was found to be successful in DCM patients [105]. Besides, studies have also concluded that the use of specific drugs that act on the particular biological pathways implicated in DCM could be highly important, especially for patients who are not responding to the conventional treatments at a satisfactory level. Nevertheless, it must be kept in mind that Levett’s (2024) review concentrates on other conditions, which takes some of the relevance away from it [106].

Yet another advanced technique modulating the gut microbiota has been applied. Researches that support the concept of gut microbiota modulation in the treatment of DCM heart function and inflammatory response has recently been published, thus marketing the possibilities of incorporating gut health management along with DCM treatment [107].

Finally, it must be noted that the theme of inflammation and immunity in DCM is being examined by researchers, as the role of inflammation and immune response in DCM is now increasingly being debated. DCM pathogenesis is suggested to be dominated by the phenomena of chronic inflammation and immune dysfunction by most scientists. The importance of the immunological characterization of patients for the successful implementation of future clinical trials can be beneficial [108]. The body of evidence that grows is the one that emphasizes the possible effectiveness of the new incoming therapies that can change the management of DCM for the better, which could shed new light on its core mechanisms and make patients’ lives better.

## 12. Conclusions

Secondary dilated cardiomyopathy represents a complex and heterogeneous condition arising from diverse extrinsic factors, including systemic diseases, toxins, and endocrinological disorders. Accurate diagnosis relies on comprehensive clinical assessment, advanced imaging techniques, and the identification of reversible causes, which can significantly influence outcomes. The therapeutic approach involves addressing the underlying condition, optimizing standard heart failure pharmacotherapy, and, when necessary, employing device-based or surgical interventions. While substantial progress has been made, further research is essential to refine diagnostic tools, explore novel therapeutic strategies, and improve outcomes for patients with secondary DCM.

## Figures and Tables

**Table 1:** Classification of secondary DCM by epidemiology (with references).

Category	Details
Regional Prevalence	Higher in tropical/less developed regions (Briceno, Schuster et al., 2016 [16])
Contribution to Heart Failure	Represents 30–50% of DCM cases (Heymans, Lakdawala et al., 2023 [9])
Developed Countries—Common Causes	Linked to hypertension, coronary artery disease (Sivitz and Nagdev, 2012 [18])
Developing Countries—Common Causes	Infectious causes like Chagas disease prevalent in Latin America
Hospitalized Patients	Accounts for 10–20% among hospitalized HF patients (Verdonschot and Heymans, 2023 [17])
Specific Risk Factors	Chemotherapy (e.g., anthracyclines), alcohol abuse, systemic inflammatory diseases, older age, male sex
Nutritional Deficiencies	Vitamin D deficiency; example from Ethiopia (Moges, Shiferaw et al., 2017 [20])
Socioeconomic Disparities	Limited access to nutrition and healthcare in low-income communities (Moges, Shiferaw et al., 2017 [20])

**Table 2:** Summary of major etiological classes of secondary dilated cardiomyopathy.

Etiological Class	Definition	Common Causes	Diagnostic Tools	Estimated Incidence
Toxic	Cardiomyopathy resulting from exposure to harmful substances, including drugs, alcohol, and chemicals.	Anthracyclines (e.g., doxorubicin), alcohol, heavy metals, illicit drugs	History of toxin exposure, ECG, echocardiography, cardiac MRI	Common in cancer patients (up to 9% for anthracyclines); varies by exposure type
Infectious	Myocardial damage due to viral, bacterial, or parasitic infections.	Coxsackievirus, Cytomegalovirus, *Trypanosoma cruzi* (Chagas disease)	Serology, cardiac MRI, endomyocardial biopsy	Significant in endemic areas (e.g., Chagas: 30% of infected individuals)
Autoimmune	Cardiac inflammation and dysfunction due to immune system attacking the myocardium.	Systemic lupus erythematosus, sarcoidosis, post-viral immune activation	Autoantibodies, inflammatory markers, CMR, biopsy	Low to moderate; varies with underlying autoimmune disease prevalence
Endocrine	Cardiomyopathy secondary to hormonal imbalances or deficiencies.	Hypothyroidism, hyperthyroidism, adrenal insufficiency	Hormonal panels (TSH, cortisol), echocardiography	Variable; hypothyroidism and adrenal insufficiency-induced DCM are rare but reversible
Peripartum	DCM occurring during the last trimester of pregnancy or postpartum period.	Pregnancy-associated hormonal changes and oxidative stress	Echocardiography, BNP levels, clinical correlation with pregnancy	Incidence ranges from 1 in 1000 to 1 in 3000 live births globally
Nutritional	Cardiomyopathy due to deficiency of key nutrients essential for cardiac function.	Vitamin D, selenium, calcium deficiencies; post-bariatric surgery malnutrition	Blood nutrient levels, echocardiography, clinical history	Rare; more frequent in low-resource settings and post-bariatric surgery patient
Chemotherapy-Induced	Cardiac dysfunction secondary to antineoplastic treatments leading to systolic impairment and ventricular dilation.	Anthracyclines, trastuzumab, cyclophosphamide, ICIs, 5-FU, bevacizumab	Cardiac MRI, biomarkers (troponin, BNP), echocardiography (strain imaging), clinical monitoring	Up to 9% in patients receiving anthracyclines; varies by agent and patient risk factors
Dyssynchronopathy (LBBB-Induced DCM)	Cardiomyopathy due to electromechanical dyssynchrony from left bundle branch block (LBBB), reversible with resynchronization.	Isolated LBBB, post-surgical conduction block	ECG, echocardiography with dyssynchrony analysis, cardiac MRI	Subset of heart failure with reduced EF; improves with CRT in selected patients
AF-Induced DCM	Tachycardia-induced cardiomyopathy due to persistent atrial fibrillation.	Chronic uncontrolled AF	ECG, Holter monitoring, echocardiography	Up to 25% of patients with AF-related heart failure show LV recovery with rhythm control
VES-Induced DCM	Cardiomyopathy due to high burden of ventricular ectopic beats; reversible after arrhythmia control.	Frequent monomorphic PVCs (>10,000/day), idiopathic or structural	ECG, Holter, echocardiography, electrophysiological study	~5% of patients with high PVC burden may develop reversible DCM

**Table 3:** Imaging techniques for diagnosing secondary dilated cardiomyopathy (DCM).

Imaging Technique	Strengths	Limitations
Transthoracic Echocardiography (TTE)	Widely available, non-invasive, real-time assessment of cardiac function and chamber size	Operator-dependent, limited by poor acoustic windows, limited tissue characterization
Cardiac Magnetic Resonance (CMR)	High-resolution imaging, tissue characterization (e.g., fibrosis), accurate volume, and EF measurements	Costly, limited availability, contraindications in patients with metal implants or severe renal dysfunction
Cardiac CT	Excellent for coronary artery visualization and calcium scoring; quick scan times	Radiation exposure, less informative for functional assessment, limited soft tissue detail
Nuclear Imaging	Assesses perfusion, metabolism, and receptor expression; useful for specific causes like sarcoidosis	Radiation exposure, lower spatial resolution, limited availability, expensive

**Table 4:** Pharmacological treatments for secondary dilated cardiomyopathy.

Therapy	Indications	Benefits	Limitations
Thyroid Hormone Replacement (Levothyroxine)	DCM due to hypothyroidism	Reduces LV size, improves ejection fraction, and may reverse DCM	Requires careful hormone monitoring and dose adjustment
Glucocorticoid Therapy (Corticosteroids)	DCM due to adrenal insufficiency	Can result in full cardiac function recovery	Diagnosis of adrenal insufficiency may be delayed or missed
Anticoagulation Therapy	Presence of ventricular thrombi or high thromboembolic risk	Reduces thromboembolic complications, improves prognosis	Risk of bleeding; requires monitoring of coagulation status
ACE Inhibitors and Beta-Blockers	Heart failure symptoms and blood pressure control in DCM	Improves symptoms, reduces mortality in heart failure	Does not address the underlying cause of secondary DCM directly

**Table 5:** Non-pharmacological therapy of secondary dilated cardiomyopathy.

Surgical Procedure	Indications	Strengths	Limitations
Mitral valve repair	Mitral insufficiency due to DCM	Improves ejection fraction, limits heart failure symptoms	Surgical risks are not suitable for all patients
Aortic valve replacement	Aortic stenosis leading to DCM	Restores cardiac function, lowers all-cause mortality	Invasive, requires thorough assessment for suitability
Heart transplant	End-stage heart failure unresponsive to other treatments	Improves survival and quality of life in end-stage DCM	Limited donor availability, immune suppression required
Catheter ablation	Atrial fibrillation/frequent PVCs	May restore sinus rhythm and reduce long-term chances of ischemic events	Surgical risks are not suitable for all patients
Cardiac resynchronization therapy (CRT)	Dyssynchronopathy due to LBBB	Restores normal electrical conduction, improves EF	Surgical risks, not suitable for all patients
